# The miR-1185-2-3p—GOLPH3L pathway promotes glucose metabolism in breast cancer by stabilizing p53-induced SERPINE1

**DOI:** 10.1186/s13046-020-01767-9

**Published:** 2021-01-28

**Authors:** Youqin Xu, Wancheng Chen, Jing Liang, Xiaoqi Zeng, Kaiyuan Ji, Jianlong Zhou, Shijun Liao, Jiexian Wu, Kongyang Xing, Zilong He, Yang Yang, Qianzhen Liu, Pingyi Zhu, Yuchang Liu, Li Li, Minfeng Liu, Wenxiao Chen, Wenhua Huang

**Affiliations:** 1 Taishan People’s Hospital, Postdoctoral Innovation Practice Base of Southern Medical University, Taishan, 529200 China; 2 National Key Discipline of Human Anatomy, School of Basic Medical Science, Southern Medical University, Guangzhou, 510515 China; 3 Affiliated Hospital of Guangdong Medical University, Guangdong Medical University, Zhanjiang, 524001 China; 4 School of Basic Medical Science, Guangzhou University of Chinese Medicine, Guangzhou, 510006 China; 5 Breast Center, Department of general surgery, Nanfang Hospital, Southern Medical University, Guangzhou, 510515 China; 6 The Eighth Affiliated Hospital, Sun Yat-sen University, Shenzhen, 518000 China; 7 Department of Radiology, Nanfang Hospital, Southern Medical University, Guangzhou, 510515 China; 8 Department of Pathology, School of Basic Medicine, Guangdong Medical University, Zhanjiang, 524023 China

**Keywords:** Tumorigenesis, Glucose metabolism, Glycosylation, miRNA, p53-induced transcription

## Abstract

**Background:**

Phosphatidylinositol-4-phosphate-binding protein GOLPH3L is overexpressed in human ductal carcinoma of the breast, and its expression levels correlate with the prognosis of breast cancer patients. However, the roles of GOLPH3L in breast tumorigenesis remain unclear.

**Methods:**

We assessed the expression and biological function of GOLPH3L in breast cancer by combining bioinformatic prediction, metabolomics analysis and RNA-seq to determine the GOLPH3L-related pathways involved in tumorigenesis. Dual-luciferase reporter assay and coimmunoprecipitation (Co-IP) were used to explore the expression regulation mechanism of GOLPH3L.

**Results:**

We demonstrated that knockdown of GOLPH3L in human breast cancer cells significantly suppressed their proliferation, survival, and migration and suppressed tumor growth in vivo, while overexpression of GOLPH3L promoted aggressive tumorigenic activities. We found that miRNA-1185-2-3p, the expression of which is decreased in human breast cancers and is inversely correlated with the prognosis of breast cancer patients, is directly involved in suppressing the expression of GOLPH3L. Metabolomics microarray analysis and transcriptome sequencing analysis revealed that GOLPH3L promotes central carbon metabolism in breast cancer by stabilizing the p53 suppressor SERPINE1.

**Conclusions:**

In summary, we discovered a miRNA-GOLPH3L-SERPINE1 pathway that plays important roles in the metabolism of breast cancer and provides new therapeutic targets for human breast cancer.

**Supplementary Information:**

The online version contains supplementary material available at 10.1186/s13046-020-01767-9.

## Background

Breast cancer is the most commonly diagnosed and most deadly cancer affecting women worldwide [1]. Tumor cells present a number of characteristics, such as self-proliferation ability [2], apoptosis resistance [3], unlimited replication potential [4], insensitivity to growth inhibition [5], continuous angiogenesis [6], tissue invasion [7] and metastasis [8]. Mammary tumorigenesis is a multistep process involving activation of oncogenes or inactivation of tumor suppressors, abnormal expression of noncoding RNA [9], loss of genome stability [10] and various genetic and epigenetic alterations [11]. Tumor cells prefer glycolysis for glucose metabolism under aerobic conditions (Warburg effect) rather than mitochondrial oxidative phosphorylation, which is more efficient for ATP production so that tumor cells can produce more energy and various metabolites in a short period of time [12]. ATP produced by glycolysis can satisfy high energy demand for rapid tumor growth. The intermediate products of glycolysis, such as glucose 6-phosphate and pyruvic acid, which can synthesize fatty acids and nucleic acids, regulate cell metabolism and biosynthesis. Moreover, glycolysis products acidify the microenvironment around the tumor, which is not conducive to the growth of normal cells but promotes tumor invasion and metastasis. The tumor suppressor protein p53 is the “guardian of the genome”, which plays critical roles in cell cycle regulation, DNA repair, cell differentiation and apoptosis [13]. Tumor cells with p53 inactivation often show increasing glycolytic activity. Accumulating data show that p53 may confer tumor suppression by inhibiting the cancer metabolic switch from oxidative phosphorylation to glycolysis.

The Golgi apparatus is an important part of the cell membrane system involved in protein glycosylation, proteolytic activation and cellular secretory activity. Protein glycosylation is one of the most common posttranslational modifications, which regulates the location, function, activity, life span and diversity of proteins in tissues and cells and participates in various important life activities, such as cell recognition, differentiation, development, signal transduction and immune response. Glycosylation labels different proteins and changes the conformation of polypeptides to increase the stability of proteins. Mammalian proteins have three types of glycosylation: N-glycosylation (N-GlcNAc), O-glycosylation (O-GlcNAc) and glycosylphosphatidylinositol (GPI) anchor. In N-glycosylation, the sugar chain is covalently linked to the free NH_2_ group of aspartic acid of the protein. The synthesis of the N-linked sugar chain starts from the endoplasmic reticulum (ER) and is completed at the Golgi. The glycoproteins formed by the ER have similar sugar chains. After entering the Golgi apparatus from the cis surface, most of the mannose on the original sugar chain is removed during the transport process between the membrane capsules. However, different types of sugar molecules are added to different glycosyltransferases to form oligosaccharide chains with different structures. N-glycan biosynthesis coordinates the cellular response of tumor cells, determining growth, invasion and drug sensitivity [14]; for instance, N-acetyllactosamine N-glycans mediate PD-L1 and PD-1, affecting the efficacy of anti-PD-L1 therapies [15]. Altering the N-glycosylation of integrins affects cis-interactions with important membrane receptors, such as EGFR, contributing to tumor cell motility and migration [16]. The N-glycosylation product Fut8 is involved in the expression of cancer biomarkers as well as in the treatment of cancer, and GnT-V is highly associated with cancer metastasis, whereas GnT-III is associated with cancer suppression [17]. O-linked glycosylation takes place in the Golgi apparatus. The sugar chain is covalently linked to the free OH radical of serine/threonine residues in proteins. O-GlcNAc is responsible for cancer progression. O-GlcNAc modifications regulate the activities of FoxM1 and cyclin D1, which are involved in cell cycle progression and critical to cell proliferation [18]. O-GlcNAc has been implicated in cancer cell survival through the effect of hyper-O-GlcNAc via activation of κB-mediated signaling [19]. Moreover, O-GlcNAc participates in cancer cell invasion and metastasis by regulating E-cadherin trafficking and function [20]. Increased levels of O-GlcNAc transferase (OGT) have been found in breast cancer [19]. O-GlcNAcylation serves as a nutrient sensor to modulate crosstalk with phosphorylation, such as p53 [21]. The GPI glycosylphosphatidylinositol anchor is the only way for proteins to combine with the cell membrane, which is different from the general lipid modifying components, and its structure is extremely complex. Mutation in the X-linked PIGA gene increases the risk of developing leukemia [22]. The spatial structure of glycoprotein determines unique glycosyltransferases to initiate certain glycosylation modifications.

Current studies show that Golgi apparatus dysfunction is associated with tumor development, but more than 80% of Golgi-related proteins have not been reported to play a role in this process. Golgi phosphoprotein 3-like (GOLPH3L) is an important protein involved in the formation of Golgi vesicles and their anterograde transport to the plasma membrane. GOLPH3L is highly expressed in various tumor tissues and promotes the proliferation of rhabdomyosarcoma cells [23] and is involved in the regulation of proliferation, apoptosis and the cell cycle in cervical cancer cells [24]. Mechanistically, GOLPH3L has been reported as an activator of the NF-κB signaling pathway in ovarian cancer [25]. Considering the critical roles of glycolysis in tumorigenesis, we demonstrate here that GOLPH3L contributes to tumorigenesis by promoting glucose metabolism in breast cancer by stabilizing certain downstream proteins of p53.

## Methods

### BRC patient samples

All patient-related studies were approved by the Institutional Review Board of Taishan People’s Hospital and Nanfang Hospital of Southern Medical University.

### Human Cancer cell Xenograft model

All animal work was approved by the Institutional Animal Care and Use Committee (IACUC) of Southern Medical University. A total of 5 × 10^6^ breast cancer cells were implanted into the skeletal muscle of the hind limbs of 3 ~ 4-week-old BALB/c nude mice (nu/nu). One week after transplantation, the diameter of tumors was measured every 3 days. Tumors were recovered and weighed after 3 weeks.

### Cell culture

Human normal mammary epithelial cell line (MCF-10A) and human breast cancer cell lines (T47D, BT474, MCF-7, MDA-MB-231, SK-BR-3) were purchased from American Type Culture Collection (ATCC, Manassas, VA, USA). Human breast cancer cell lines (T47D, BT474, MCF-7, MDA-MB-231, SK-BR-3) were cultured in Roswell Park Memorial Institute 1640 medium (Gibco, USA) supplemented with 10% fetal bovine serum (FBS, HyClone, Utah, USA) and 1% penicillin-streptomycin (Pen/Strep) (Gibco) at 37 °C with 5% CO_2_. The human normal mammary epithelial cell line MCF-10A was cultured in media and supplements from the MEGM kit (Lonza/Clonetics, CC-3150) and 10% FBS - supplemented with 100 ng/ml cholera toxin (Sigma-Aldrich, C8052) at 37 °C with 5% CO_2_.

### Establishment of transfected cell lines

The vectors expressing GOLPH3L-specific siRNA (RIBOBIO, Cat# siG000055204A-C) and SERPINE1-specific siRNA (ThermoFisher, Cat# 4390771). Vectors expressing human GOLPH3L cDNA and SERPINE1 cDNA were transfected into cells as previously described [26]. Cells were selected with puromycin (2 μg/ml, GeneChem) for 3 days for stable transfection.

### Western blot analysis

Cells were extracted for total protein analysis using lysis buffer, and samples were separated on 8–15% SDS-PAGE and transferred to nitrocellulose membranes, which were blocked with blocking buffer (5% skim milk in PBS with 0.05% Tween 20) and incubated with primary antibody in the blocking buffer. After being washed three times with blocking buffer, the membrane was probed with secondary antibody and developed with Supersignal West Pico or Dura (Thermo Fisher Scientific).

### Quantitative PCR analysis

Real-time PCR analysis was performed using the StepOnePlus Real-Time PCR System (Applied Biosystems) with FastStart Universal SYBR Green Master Mix (Roche) as previously reported. The primer sets used were as follows:

- GAPDH-F: GAACGGGAAGCTCACTGG;
- GAPDH-R: GCCTGCTTCACCACCTTCT;
- GOLPH3L-F: GTAAATGACCCTCAGCGTATGG;
- GOLPH3L-R: GTTCTACTAAGTCCTTGGCTCGAT;
- miRNA-1185-2-3p: AUAUACAGGGGGAGACUCUCAU;
- miRNA-1185-2-3p-F: ACACTCCAGCTGGGATATACAGGGGGAGAC;
- miRNA-1185-2-3p-R: CTCAACTGGTGTCGTGGA;
- U6-F: GCTTCGGCAGCACATATACTAAAAT;
- U6-R: CGCTTCATGAATTTGCGTGTCAT;
- miRNA-1185-2-3p mimic-F: AUAUACAGGGGGAGACUCUCAU;
- miRNA-1185-2-3p mimic-R: GAGAGUCUCCCCCUGUAUAUUU;
- miRNA-1185-2-3p inhibitor: AUGAGAGUCUCCCCCUGUAUAU.
- SERPINE1-F: ACCGCAACGTGGTTTTCTCA;
- SERPINE1-R: TTGAATCCCATAGCTGCTTGAAT.

The PCR conditions were as follows: 10 min at 95 °C, 40 cycles of 15 s at 95 °C, and 1 min at 60 °C. The average Ct value for each gene was determined from triplicate reactions and normalized to that of β-actin for genes and U6 for microRNAs (miRNAs).

### Cell proliferation, apoptosis, migration assay and cell cycle assay

For EdU-high content screening of the cellular proliferation assay, cells were prepared by trypsinization and seeded onto 96-well plates at a density of 1 × 10^4^ cells per well. After incubation for 48 h at 37 °C, the old medium was discarded, and 100 μl of medium containing EdU was added to each well and incubated for 6 h. Cells were fixed with paraformaldehyde at room temperature for 20 min. Then, 100 μl of 2 mg/ml glycine solution and 100 μl of 0.5% Triton X-100 solution were added to the wells separately, and the cells were washed twice with PBS. One hundred microliters of Apollo-643 staining solution was added in each well for 30 min and then discarded. After destaining and rinsing, DAPI reaction mixture was added to each well and incubated for 30 min. A GE IN CELL Analyzer 6500HS Confocal High Content Imaging Analyzer was used for collecting images.

Consistent treatments were applied to prepared cells in 96-well plates: 100 μl 2 mg/ml glycine solution and 100 μl 0.5% Triton X-100 solution were added into wells separately, and then the cells were washed twice with PBS. Fluorescein-dUTP solution (50 μl) mixed with TdT enzyme was added to each well and incubated for 2 h at room temperature. Then, the reaction solution was discarded, and 100 μl 2 × SSC buffer was immediately added into each well. A 1× DAPI reaction mixture was added to the plates and incubated for 30 min. A GE IN CELL Analyzer 6500HS Confocal High Content Imaging Analyzer was used for collecting images after the cells were washed three times with PBS.

Consistent treatments were applied to prepared cells in 24-well plates at a density of 2.5 × 10^4^ cells per well. After adjusting the cell concentration to 1 × 10^5^ cells/ml, the cells were seeded into the upper chamber at 1 × 10^4^ cells/μl, and the lower chamber was immediately filled with 150 μl complete medium with 10% FBS as a chemoattractant and then incubated for 48 h. After the medium was discarded from the lower chamber, 150 μl PBS was added to each well for 5 min. Calcein AM cell staining solution at a working concentration of 2.5 μM was prepared with complete medium, and 150 μl staining solution was added to each well for incubation at room temperature for 30 min after the PBS was discarded. After incubation, 150 μl trypsin was added to the lower chamber and incubated at 37 °C for 15 min. Serum-containing medium was added to the chamber to stop digestion. Cells in the subchamber were aspirated for cell counting.

For the cell cycle assay, cells were prepared in 96-well plates at a density of 1 × 10^4^ cells per well. After being washed with PBS one time, the cells were fixed with 4% paraformaldehyde for 20 min; the paraformaldehyde was removed, and 100 μl of 2 mg/ml glycine solution was added. The glycine solution was subsequently removed, and the cells were washed with PBS one time. Then, 100 μl of 0.5% Triton X-100 PBS solution was added to each well, which were then washed twice with PBS. One hundred microliters of 1× DAPI reaction mixture was added to each well, and the plates were incubated for 30 min. After being washed with PBS three times, a GE IN CELL Analyzer 6500HS Confocal High Content Imaging Analyzer was used to collect the images.

### Dual-luciferase reporter assay

Cells were seeded in triplicate onto 6-well plates at a density of 4 × 10^5^ cells/well for 48 h and transfected with 0.3 μg of REPOTM-AP-1-luc plasmid and control-luciferase plasmid separately together with 30 ng of pGMR TK renilla plasmid (GenomeDitech, Shanghai, China) using Lipofectamine™ 3000 reagent (Invitrogen, Carlsbad, USA). Firefly and Renilla luciferase activities were measured using the Dual-Luciferase Reporter Assay Kit (Promega, Madison, USA) after 48 h of transfection.

### Metabolomic analysis

Cells were collected in centrifuge tubes filled with 1 ml methanol:acetonitrile:water (2:2:1, V/V) at a density of 2 × 10^7^ cells/tube after washing with PBS and physiological saline solution. The liquid nitrogen is stored at − 80 °C after quick freezing. To analyze metabolomics, samples were analyzed at Applied Protein Technology (APT, Shanghai). Partial least squares discrimination analysis was applied to reveal the relationship between the expression of metabolites and sample types by calculating the cast variable importance for the projection (VIP) to measure the expression pattern of each metabolite for each group. All differentially expressed metabolites were selected for Gene Ontology (GO) and Kyoto Encyclopedia of Genes and Genomes (KEGG) pathway analyses (VIP score > 1.0). GO was performed with KOBAS3.0 software. The differentially expressed metabolites and enriched pathways were mapped using the KEGG pathways with KOBAS3.0 software (http://www.genome.jp/kegg).

### Transcriptome sequencing

Total RNA was isolated from cells/tissues using TRIzol (Invitrogen) according to the manufacturer’s protocol. RNA purity was assessed using the ND-1000 Nanodrop. Each RNA sample had an A260:A280 ratio above 1.8 and an A260:A230 ratio above 2.0. RNA integrity was evaluated using the Agilent 2200 TapeStation (Agilent Technologies, USA), and each sample had an RNA integrity number (RIN) above 7.0. Briefly, rRNAs were removed from total RNA using the EpicentreRibo-Zero rRNA Removal Kit (Illumina, USA) and fragmented to approximately 200 bp. Subsequently, the purified RNAs were subjected to first strand and second strand cDNA synthesis followed by adaptor ligation and enrichment with a low-cycle according to the instructions of the NEBNext® Ultra™ RNA Library Prep Kit for Illumina (NEB, USA). The purified l library products were evaluated using the Agilent 2200 TapeStation and Qubit®2.0 (Life Technologies, USA) and then diluted to 10 pM for cluster generation in situ on the pair-end flow cell followed by sequencing (2 × 150 bp) with a HiSeq3000. Clean reads were obtained after removal of reads containing adaptor, poly-N and low quality from raw data. HISAT2 was used to align the clean reads to the mouse reference genome mm10 with default parameters. HTSeq was subsequently employed to convert aligned short reads into read counts for each gene model. Differential expression was assessed by DEseq using read counts as input. The Benjamini-Hochberg multiple test correction method was enabled. Differentially expressed genes were chosen according to the criteria of fold change > 2 and adjusted *p*-value < 0.05. All the differentially expressed genes were used for heat map analysis and KEGG ontology enrichment analyses. For KEGG enrichment analysis, a *p*-value < 0.05 was used as the threshold to determine significant enrichment of the gene sets.

### Seahorse assay

To measure the levels of glycolytic ATP production, 1 × 10^4^ cells were seeded into each well of black 96-well plates. To normalize the levels of protein, the same number of cells were seeded into clear bottom 96-well plates. Cells were incubated in medium containing 1 μM oligomycin (Sigma-Aldrich) to inhibit mitochondrial oxidative ATP production or 25 mM 2-deoxy-D-glucose (2-DG) to inhibit glycolytic ATP production. After washing the cells with PBS, ATP levels were measured using a kit according to the manufacturer’s protocol (PerkinElmer). Total ATP production was calculated by subtracting the amount of ATP in cells treated with both oligomycin and 2-DG from the amount of ATP in cells without treatment. To normalize the number of cells, the protein concentration was measured using the Bicinchoninic Acid Protein Assay Kit (BCA, Sigma-Aldrich) according to the manufacturer’s protocols.

### Protein stability analysis

Cells were transfected with siRNAs, and cyclohexamide (CHX; 1:1000) was used to treat cells, which were harvested at various time points (0 h, 0.5 h, 1 h, 2 h, 4 h, 6 h and 12 h). Levels of various proteins were determined by Western blot analysis and quantified with ImageJ software.

### Coimmunoprecipitation assay

Immunoprecipitation assays were performed as previously described [27]. Briefly, cells were lysed in RIPA duffer containing protease and phosphatase inhibitors, and cells were collected after centrifugation at 12,000×g for 10 min at 4 °C. Supernatants were immunoprecipitated with antibodies followed by incubation with magnetic protein A/G beads (Pierce) for 2 h at 4 °C. The immune complexes were washed three times with PBS, resuspended in SDS-PAGE buffer and assessed by Western blot analysis.

### Statistical analysis

Data were analyzed using SPSS 20.0. and two-tailed independent Student’s t-test; *P* < 0.05 was considered significant. Two patient cohorts were compared by Kaplan-Meier survival plot, and log-rank *p*-values were calculated.

## Results

### GOLPH3L is highly expressed in breast Cancer and promotes the tumorigenesis of breast Cancer cells

**Fig. 1:**
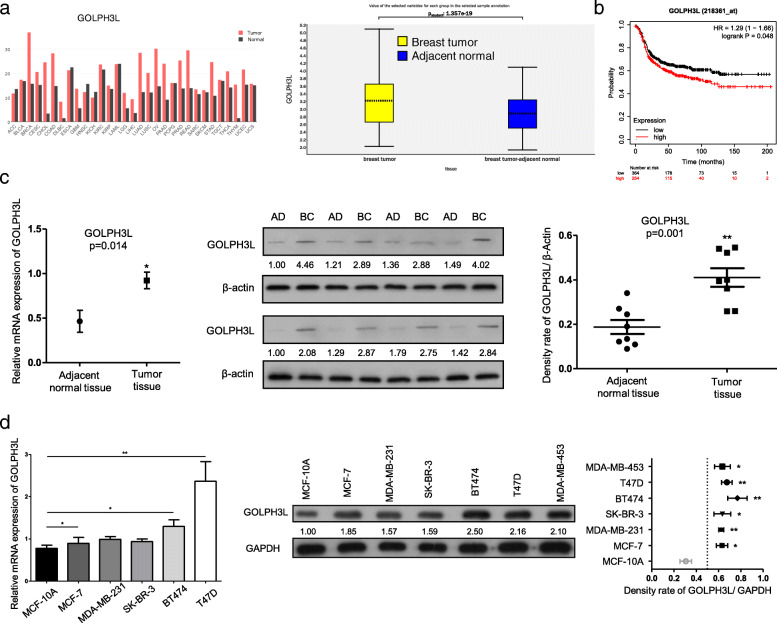
GOLPH3L is highly expressed in breast cancer and is inversely correlated with prognosis. **a** GEPIA analysis of the expression of GOLPH3L in 31 different kinds of cancer tissues and adjacent normal tissues (left panel). The comparison of GOLPH3L in the database GSE93601, containing human breast cancers (*n* = 602) and tumor-adjacent normal tissues (*n* = 508) (right panel). **b** Log-rank (Mantel Cox) survival test of breast cancer patients based on the levels of GOLPH3L mRNA (low expression *n* = 364, high expression *n* = 254). *P*-values are indicated. **c** The mRNA and protein levels of GOLPH3L were higher in breast cancer tissue samples than in adjacent normal tissues, 8 pairs, * *p* < 0.05, ** *p* < 0.01. **d** The mRNA and protein levels of GOLPH3L were higher in breast cancer cell lines than in normal mammary epithelial cells; *n* = 3, * *p* < 0.05, ** *p* < 0.01

Using Gene Expression Profiling Interactive Analysis (GEPIA, http://gepia.cancer-pku.cn/index.html) and profiling the expression data of GOLPH3L in breast tumors and tumor-adjacent normal tissues in the database (GSE93601), we confirmed that GOLPH3L was dramatically overexpressed in breast cancer samples compared to normal control samples (Fig. 1a). Moreover, the expression levels of GOLPH3L were inversely correlated with the prognosis of human breast cancer patients (Fig. 1b). In this context, GOLPH3L promotes breast tumorigenesis. To explore the explicit roles of GOLPH3L in breast tumorigenesis, we investigated the mRNA and protein expression levels of GOLPH3L in breast cancer tissues and paired adjacent normal tissues as well as in various breast cancer cell lines and normal breast epithelial cell lines. The relative mRNA and protein expression of GOLPH3L in cancer tissues was much higher than that in adjacent normal tissues (Fig. 1c). Consistently, compared to the normal breast epithelial cell line MCF-10A, the expression levels of GOLPH3L mRNA and protein were increased in breast cancer cell lines, especially in T47D and BT474 cells (Fig. 1d).

**Fig. 2:**
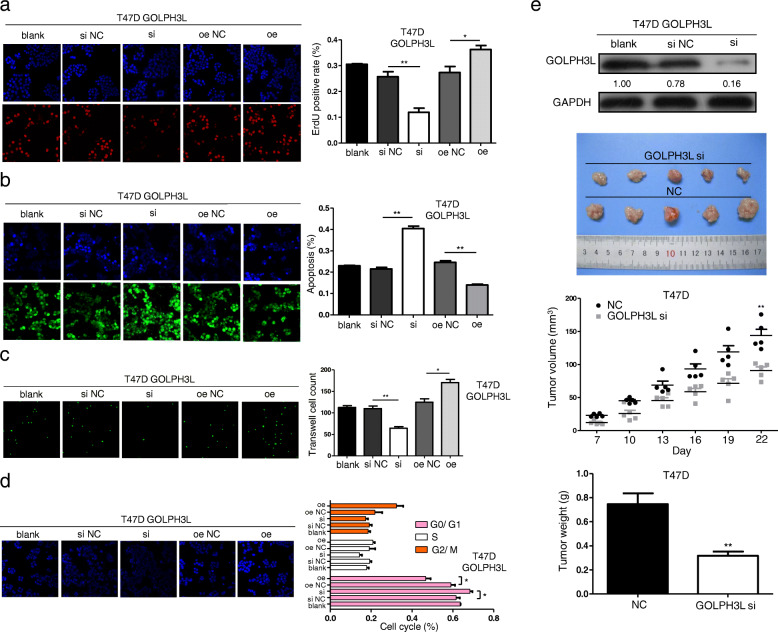
GOLPH3L promotes tumorigenesis of breast cancer cells. **a** The proliferation of T47D cells before and after GOLPH3L alteration. The cell number was determined with EdU-high content screening in the cellular proliferation assay; *n* = 3, * *p* < 0.05, ** *p* < 0.01. **b** Knockdown and overexpression of GOLPH3L in T47D cells affected apoptosis. The cells were counted with a fluorescein assay; *n* = 3, * *p* < 0.05, ** *p* < 0.01. **c** Altering the expression of GOLPH3L inhibited the migration of T47D cells using a Transwell assay; *n* = 3, * *p* < 0.05, ** *p* < 0.01. **d** The knockdown of GOLPH3L significantly blocked the cell cycle at the G0/G1 phase according to the fluorescein assay at OD 405 nm; *n* = 3, * *p* < 0.05. **e** Analysis of the protein level of GOLPH3L in T47D cells before and after silencing (top panel). Suppressing the expression of GOLPH3L inhibited the tumor growth of T47D cells in nude mice, *n* = 5, ** *p* < 0.01 (three bottom panels)

Therefore, we examined the roles of GOLPH3L in T47D and BT474 cells by altering the expression of GOLPH3L through knockdown or overexpression (Figure S1a and b). Restraining GOLPH3L expression in breast cells decreased cellular proliferation and survival, while upregulating the expression of GOLPH3L in T47D cells promoted proliferation and survival (Fig. 2a and b). Transwell assays revealed that silencing GOLPH3L inhibited the migration of T47D cells, and in contrast, overexpressing GOLPH3L in cells obviously facilitated cancer cell migration (Fig. 2c). Moreover, knockdown of GOLPH3L expression significantly blocked the cell cycle at the G0/G1 phase (Fig. 2d). Further evidence of the role of GOLPH3L in tumorigenesis is that knockdown of GOLPH3L in T47D cells significantly reduced the growth of tumors formed by T47D cells in immunodeficient mice (Fig. 2e). Consistent data were obtained using BT474 breast cancer cells (Figure S1c-f). These findings demonstrate that GOLPH3L promotes the tumorigenesis of breast cancer cells in various ways.

### miRNA-1185-2-3p negatively regulates GOLPH3L and positively correlates with the prognosis of human breast Cancer patients

**Fig. 3:**
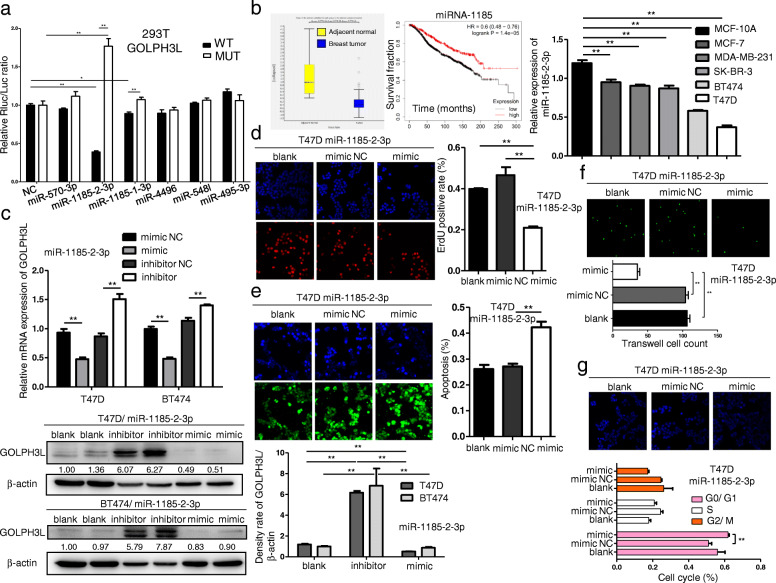
miR-1185-2-3p directly inhibits the expression of GOLPH3L and suppresses tumorigenic activities. **a** miR-1185-2-3p targeted the predicted sites within the 3′-UTR of GOLPH3L, and mutation of the predicted target sites rescued the inhibitory effects of miR-1185-2-3p; *n* = 3, * *p* < 0.05, ** *p* < 0.01. **b** The comparison of the levels of miR-1185-2-3p in human breast cancers (*n* = 101) and tumor-adjacent normal tissues (*n* = 15) with GSE45666 (left panel). Log-rank (Mantel Cox) test of breast cancer patient survival based on the levels of miR-1185-2-3p (low expression *n* = 868, high expression *n* = 394); *p*-values are indicated (middle panel). The expression of miR-1185-2-3p was significantly reduced in various breast cancer cells; *n* = 3, ** *p* < 0.01 (right panel). **c** Inhibition and overexpression of miR-1185-2-3p inversely correlated with the levels of GOLPH3L mRNA (top panel) and protein (bottom panel); *n* = 3, ** *p* < 0.01. d Overexpression of miR-1185-2-3p reduced the proliferation of T47D cells; *n* = 3, ** *p* < 0.01. **e** miR-1185-2-3p overexpression significantly induced apoptosis in T47D cells; *n* = 3, ** *p* < 0.01. **f** Overexpression of miR-1185-2-3p reduced the migration of T47D cells; *n* = 3, ** *p* < 0.01. **g** Overexpression of miR-1185-2-3p blocked the cell cycle at G0/G1 phase; *n* = 3, ** *p* < 0.01

miRNAs are 19–25 nucleotide-long noncoding RNAs that regulate gene expression via messenger RNA degradation and translation. miRNAs are involved in every aspect of biological processes, and it is worth noting that alterations in miRNA expression induce the initiation, progression and metastasis of human tumors. Considering the consequence of GOLPH3L in promoting breast tumorigenesis, we utilized the miRDB prediction program (http://mirdb.org/). Based on the predicted results (Figure S2a), we mutated the target sites of the top five predicted miRNAs in the 3′-UTR of GOLPH3L mRNA. A dual-luciferase reporter assay confirmed that miRNA-1185-2-3p regulated the levels of GOLPH3L mRNA by targeting the predicted sites within the 3′-UTR of GOLPH3L (Fig. 3a). Based on the GEO dataset GSE45666, we found that miRNA-1185-2-3p was expressed at a higher level in tumor-adjacent normal tissues than in breast tumor tissues and was inversely correlated with the prognosis of breast cancer patients (Fig. 3b, left panel). Consistently, the expression of miRNA-1185-2-3p was higher in various breast cancer cell lines than in the normal breast epithelial cell line MCF-10A (Fig. 3b, right panel). To determine the impact of miRNA-1185-2-3p on the expression of GOLPH3L, we used miRNA mimics and inhibitors to demonstrate that the expression levels of miRNA-1185-2-3p were inversely correlated with the expression of GOLPH3L in breast cancer lines (Fig. 3c and S2b). These results support the notion that miRNA-1185-2-3p directly targets GOLPH3L mRNA.

Since miRNA-1185-2-3p suppresses the expression of GOLPH3L in breast cancer cells, we induced it in the breast cancer cell lines T47D and BT474 to determine whether miRNA-1185-2-3p could have the same tumor suppressive effects as GOLPH3L knockdown. The overexpression of miRNA-1185-2-3p also inhibited proliferation, survival, and migration and arrested the cell cycle of breast cancer cells (Fig. 3d-g and S2c-f). In addition, the induction of miRNA-1185-2-3p suppressed the tumorigenesis induced by GOLPH3L overexpression (Figure S3). Therefore, these data indicate that miRNA-1185-2-3p could inhibit breast tumorigenesis by suppressing the expression of GOLPH3L.

### GOLPH3L affects glucose metabolism in breast Cancer cells

**Fig. 4:**
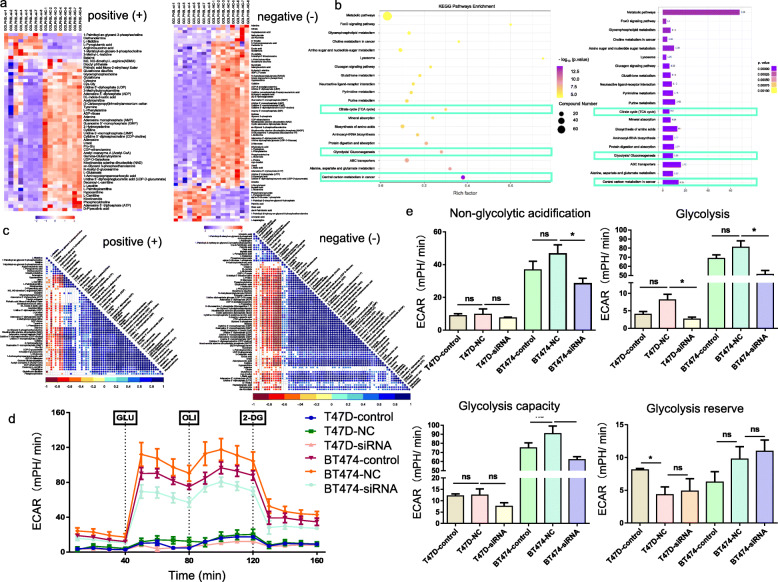
GOLPH3L promotes glucose metabolism in breast cancer cells. **a** Heat map of significantly altered metabolites after GOLPH3L suppression in the positive ion mode (left panel) and negative ion mode (right panel); *p*-values< 0.05 are indicated. **b** Bubble diagram of KEGG pathways related to the inhibition of GOLPH3L (left panel) and GO analysis of pathways affected by GOLPH3L silencing (right panel); *p*-values< 0.05 are indicated. **c** Correlation between different metabolites altered by the expression of GOLPH3L in the positive ion mode (top panel) and negative ion mode (bottom panel); *p*-values< 0.05 are indicated. **d** and **e** ECAR in cells of the control groups and GOLPH3L-knockdown group transfected with empty vector or siRNA in response to glucose, oligomycin and 2-DG. Data are presented as the mean value ± SD, two-way ANOVA with Tukey’s multiple comparison test, *n* = 3. Independent experiments for each group, *p*-values< 0.05 were indicated

To understand the mechanism by which GOLPH3L promotes tumorigenesis, we combined metabolomic analysis and transcriptome sequencing in GOLPH3L-silenced T47D breast cancer cells. We identified metabolites that were altered significantly after GOLPH3L silencing. Notably, glycolytic intermediates such as acetyl coenzyme A (acetyl-CoA), adenosine monophosphate (AMP), adenosine 5′-diphosphate (ADP) and adenosine 5′-triphosphate (ATP) were reduced when GOLPH3L expression was downregulated (Fig. 4a, S4a and Table 1). KEGG pathway analysis showed that GOLPH3L expression could markedly influence central carbon metabolism in cancer, glycolysis/gluconeogenesis and 61 other pathways (Fig. 4b and Table 2). Correlation analysis of different metabolites revealed that acetyl-CoA, AMP, ADP and ATP were positively correlated in both positive ion mode (Fig. 4c, top panel) and negative ion mode (Fig. 4c, bottom panel). Since GOLPH3L knockdown decreased the proliferation of breast cancer cells, we evaluated the impact of GOLPH3L on metabolism and found that the suppression of GOLPH3L expression decreased glycolytic activity in breast cell lines (Fig. 4d and e).

**Table 1:** Metabolites that were altered significantly after GOLPH3L silencing

Name	ID	VIP	Fold change	***p***-value
M348T450	Adenosine monophosphate (AMP)	5.35017309	0.343953619	1.85022E-09
M489T438	Cytidine 5′-diphosphocholine (CDP-choline)	3.769023161	0.294683065	2.09147E-09
M325T436	Uridine 5′-monophosphate (UMP)	2.859910755	0.36416908	1.85344E-08
M136T425	Adenine	3.624555449	0.541578913	3.52988E-08
M216T387	sn-Glycerol 3-phosphoethanolamine	3.817810077	0.662660731	1.64313E-07
M113T160	Uracil	2.339930939	0.254999039	2.66289E-07
M162T355_2	L-carnitine	2.259917766	0.687860774	5.55508E-07
M244T235	Cytidine	1.320380286	0.343617832	6.16343E-07
M664T430	Nicotinamide adenine dinucleotide (NAD)	5.426903613	0.531267518	6.8003E-07
M204T426	N-acetyl-D-glucosamine	2.162566187	0.585855735	6.80877E-07
M268T167	Adenosine	2.080090077	0.506337679	8.19558E-07
M152T457	2-Hydroxyadenine	1.192073219	0.418366736	1.00101E-06
M364T457	Guanosine 5′-monophosphate (GMP)	2.20531908	0.381298814	1.34425E-06
M598T474	Uridine 5′-diphosphoglucuronic acid (UDP-D-glucuronate)	1.241389474	0.627904863	2.896E-06
M810T418	Acetyl coenzyme A (Acetyl-CoA)	1.148074386	0.537400888	7.08625E-06
M148T391	L-glutamate	3.853584751	0.737242788	8.94063E-06
M584T438	UDP-D-galactose	1.033843982	0.641425356	9.98778E-06
M137T166	Hypoxanthine	6.446185931	0.11746834	1.93784E-05
M428T452_2	Adenosine 5′-diphosphate (ADP)	7.163025043	0.57751916	2.09404E-05
M447T442	CDP-ethanolamine	1.026217107	0.611693587	2.16949E-05
M291T464	Argininosuccinic acid	1.026399168	1.525518377	2.31077E-05
M188T257	DL-indole-3-lactic acid	1.240956561	0.489217809	2.34219E-05
M102T391	1-Aminocyclopropanecarboxylic acid	1.410081315	0.756256635	2.38876E-05
M246T242	2-Methylbutyroylcarnitine	7.369334193	0.509326233	3.52739E-05
M106T299	Diethanolamine	1.53304285	14.44114449	3.92078E-05
M245T409	Pro-Glu	1.083816026	0.642279716	4.4156E-05
M316T193	Decanoyl-L-carnitine	1.705457563	0.620089802	8.50963E-05
M204T303	Acetylcarnitine	10.77036712	0.725339373	0.000102368
M422T466	Uridine 5′-diphosphate (UDP)	1.109528766	0.668781099	0.000142935
M400T159_2	L-palmitoylcarnitine	8.34588634	0.644174553	0.000177908
M147T369	L-pyroglutamic acid	1.765274591	1.542951135	0.000179981
M233T395	Gamma-glutamylcysteine	1.388401872	0.540837694	0.000320962
M112T236	Cytosine	1.593691839	0.555964705	0.000415561
M542T430	ADP-ribose	1.028412857	0.622601002	0.00064559
M308T428	Glutathione	4.266509616	0.504989177	0.000716522
M123T61	Nicotinamide	1.851347574	0.566997397	0.000943254
M156T441	L-histidine	1.2602915	1.504208252	0.00100569
M170T397	3-Methyl-L-histidine	2.097791259	7.382253419	0.001046756
M120T256	Tyramine	1.693843786	0.657319643	0.001442055
M166T256	L-phenylalanine	1.120111864	0.675428936	0.002216965
M146T374_2	(3-Carboxypropyl) trimethylammonium cation	3.995126207	0.750604189	0.002386475
M132T263	L-leucine	1.432367955	0.424002729	0.002807843
M179T427	Cys-Gly	1.127437605	0.643923687	0.006537627
M258T457	Glycerophosphocholine	1.162325024	0.70923693	0.018142168
M508T581	Adenosine 5′-triphosphate (ATP)	1.252149198	0.44136226	0.018216171
M391T33	Dioctyl phthalate	5.057833924	0.806525311	0.019249997
M613T491	Glutathione disulfide	2.467281297	0.722876989	0.059115948
M468T200	1-Myristoyl-sn-glycero-3-phosphocholine	1.668722344	2.054758707	0.059687763
M118T462	Betaine	2.078707794	1.308639016	0.05975974
M184T566	Phosphorylcholine	1.969089209	0.217723196	0.060512883
M496T166	1-Palmitoyl-sn-glycero-3-phosphocholine	3.467532932	1.405433036	0.060816012
M203T505	NG,NG-dimethyl-L-arginine (ADMA)	1.02231111	0.612169445	0.079805836
M130T542	D-pipecolinic acid	1.22345702	0.462637578	0.081203784
M279T33	Phthalic acid mono-2-ethylhexyl ester	2.038184914	0.869293803	0.088733314
M341T395	maltose	2.111953392	0.028561128	3.10543E-17
M347T439	Inosine 5′-monophosphate (IMP)	3.625592991	0.301881382	6.71817E-16
M322T450	Cytidine 5′-monophosphate (CMP)	1.192275542	0.62674391	2.23826E-14
M662T429	Nicotinamide adenine dinucleotide (NAD)	5.135975169	0.567384075	4.63725E-13
M547T437	Cytidine 5′-diphosphocholine (CDP-choline)	1.791062321	0.406528182	1.40724E-12
M362T455	Guanosine 5′-monophosphate (GMP)	3.653583959	0.407242856	4.41861E-12
M540T428	Cyclic adenosine diphosphate ribose	4.068411598	0.566724985	6.8843E-12
M606T424	UDP-N-acetylglucosamine	18.47592875	0.611830043	7.85683E-12
M323T434	Uridine 5′-monophosphate (UMP)	7.171175055	0.404982084	1.33639E-11
M346T422	Adenosine monophosphate (AMP)	14.34548448	0.318588959	2.04199E-11
M203T254	L-tryptophan	2.120508327	0.500782842	3.29102E-11
M214T388	sn-Glycerol 3-phosphoethanolamine	9.759185833	0.725105379	3.90018E-11
M242T234	Cytidine	1.768227149	0.351697024	1.48152E-10
M445T440	CDP-ethanolamine	2.832548838	0.60943293	2.32647E-10
M808T416	Acetyl coenzyme A (acetyl-CoA)	1.321582442	0.559526257	3.85093E-10
M742T485	Nicotinamide adenine dinucleotide phosphate (NADP)	1.711544078	0.624512533	8.56497E-10
M130T260	L-Isoleucine	3.729655739	0.588743745	1.07604E-09
M613T434	Cytidine monophosphate N-acetylneuraminic acid	3.365736279	0.515824367	1.14679E-09
M171T383	Glycerol 3-phosphate	3.123463887	0.690487464	1.17292E-09
M147T385	(S)-2-Hydroxyglutarate	2.209506147	0.672217978	1.29056E-09
M167T451	Phosphoenolpyruvate	3.418641375	1.879691109	1.38673E-09
M174T387_2	N-Acetyl-L-aspartic acid	5.248401418	0.659295634	2.85219E-09
M258T410	D-Glucosamine 1-phosphate (glucosamine-1P)	1.215448728	0.595730278	2.98007E-09
M135T165	Hypoxanthine	2.83515564	0.155797031	3.3521E-09
M565T436	Uridine diphosphate glucose (UDP-D-glucose)	4.94082239	0.593931258	5.65669E-09
M239T387	D-Mannose	1.267493145	0.837341404	1.65544E-08
M131T253	Ethylmalonic acid	1.942789906	0.670553701	2.25702E-08
M182T474	Phosphorylcholine	1.592146407	0.776441511	5.19225E-08
M426T481	Adenosine 5′-diphosphate (ADP)	2.142482016	0.786728943	6.27755E-08
M403T463	Uridine 5′-diphosphate (UDP)	6.023335277	0.590303313	6.92096E-08
M164T253	L-phenylalanine	2.745272587	0.649759377	1.09694E-07
M128T370_1	L-pyroglutamic acid	1.084478181	1.326574096	1.35422E-07
M259T448	Alpha-D-glucose 1-phosphate	1.955748269	1.727049692	6.43531E-07
M303T435	N-acetylaspartylglutamate (NAAG)	1.092531363	0.622574707	1.04037E-06
M145T372	L-glutamine	4.84006701	1.691397723	1.33369E-06
M117T380_2	Succinate	3.22018177	0.745112292	1.33821E-06
M146T390_2	L-glutamate	6.383368133	0.805813766	1.34212E-06
M111T83	Uracil	4.093084571	0.338717051	1.98724E-06
M255T38	Palmitic acid	15.91857778	1.482478443	2.63587E-06
M462T486	Adenylsuccinic acid	1.400896234	0.601445092	3.04252E-06
M179T387_2	Myo-Inositol	1.869509045	0.876085103	3.53295E-06
M588T446	GDP-L-fucose	1.036473053	0.727980689	3.8859E-06
M458T140	L-palmitoylcarnitine	1.71058671	0.69922387	3.95794E-06
M296T70	S-methyl-5′-thioadenosine	2.695515248	0.562035793	5.0904E-06
M114T308	L-Proline	1.492638552	0.844853133	6.29786E-06
M130T386	N-acetyl-L-alanine	1.284618775	0.714233544	7.07193E-06
M611T488	Glutathione disulfide	6.326051929	0.673543892	2.60415E-05
M89T221_2	DL-lactate	6.180555782	0.771109869	3.15076E-05
M506T481	Adenosine 5′-triphosphate (ATP)	6.265508138	0.776594367	7.93354E-05
M133T396	L-malic acid	3.710482706	0.870781902	0.00015885
M306T394	Glutathione	6.846802373	0.645572726	0.000202429
M452T199	1-Palmitoyl-2-hydroxy-sn-glycero-3-phosphoethanolamine	2.364541907	1.17565739	0.000288876
M281T38	Oleic acid	10.56865128	1.261282292	0.000386302
M337T38	Erucic acid	1.462001645	0.804834608	0.000563427
M483T490	Uridine 5′-triphosphate (UTP)	2.985361942	0.791767071	0.000765923
M673T189	1-Palmitoyl-2-oleoyl-sn-glycero-3-phosphate	1.958401653	1.956770427	0.001001254
M253T38	Cis-9-palmitoleic acid	3.035713741	1.176091575	0.004203955
M227T150	Myristic acid	1.009572648	1.48607569	0.004811555
M197T38	3-Hydroxydodecanoic acid	2.666768268	0.71831232	0.006944812
M333T125	Penicillin G	1.82556849	0.584544716	0.009905013
M273T34	Salicylamide	1.383050648	0.362734521	0.009978679
M579T469	Uridine 5′-diphosphoglucuronic acid (UDP-D-glucuronate)	3.315658544	0.86375998	0.010231094
M141T353	2-Oxoadipic acid	11.40052894	0.912787301	0.015048419
M130T344	Creatine	1.009533641	0.795141329	0.017407461
M154T411	Urocanic acid	1.176119208	1.475399848	0.020677476
M173T432	Cis-Aconitate	1.169850112	0.768659387	0.021442597
M219T2	4-Nonylphenol	2.042585348	1.263228143	0.043438355
M134T153	Adenine	1.249418803	0.62240901	0.043527968
M269T136	Heptadecanoic acid	1.030910387	0.689149164	0.05747134
M131T375	L-asparagine	1.700684605	1.62285133	0.061339629
M191T497	Citrate	2.212863083	0.850809508	0.067218448
M181T296	D-threitol	1.061736173	0.563835355	0.079699611

Legend: Untargeted metabolomics sequencing was used to reveal metabolites that were altered significantly after GOLPH3L silencing, and those with *p*-values< 0.05 are indicated

**Table 2:** KEGG pathway analysis of different metabolites that were altered significantly after GOLPH3L silencing

Map-ID	Map name	Ref_ per	***P***-value	FDR	Rich factor
map 05230	Central carbon metabolism in cancer	0.902218971	1.52023E-15	1.9611E-13	0.378378378
map 00250	Alanine, aspartate and glutamate metabolism	0.682760302	1.32499E-09	8.54619E-08	0.321428571
map 02010	ABC transporters	3.340648622	4.36608E-09	1.6293E-07	0.116788321
map 00010	Glycolysis/Gluconeogenesis	0.780297488	5.05208E-09	1.6293E-07	0.28125
map 04974	Protein digestion and absorption	1.146061936	1.30029E-08	3.35475E-07	0.212765957
map 00970	Aminoacyl-tRNA biosynthesis	1.267983419	4.83446E-07	1.03941E-05	0.173076923
map 01230	Biosynthesis of amino acids	3.121189954	7.70926E-07	1.42071E-05	0.1015625
map 04978	Mineral absorption	0.707144599	9.11103E-07	1.46915E-05	0.24137931
map 00020	Citrate cycle (TCA cycle)	0.48768593	1.42224E-06	2.03854E-05	0.3
map 00230	Purine metabolism	2.316508169	1.61025E-06	2.07723E-05	0.115789474
map 00240	Pyrimidine metabolism	1.584979273	3.42162E-06	4.01262E-05	0.138461538
map 04080	Neuroactive ligand-receptor interaction	1.267983419	5.53193E-06	5.94683E-05	0.153846154
map 00480	Glutathione metabolism	0.926603267	6.39595E-06	6.34675E-05	0.184210526
map 04922	Glucagon signaling pathway	0.633991709	7.69409E-06	7.08956E-05	0.230769231
map 04142	Lysosome	0.097537186	2.8207E-05	0.00024258	0.75
map 00520	Amino sugar and nucleotide sugar metabolism	2.633504023	3.71809E-05	0.000299771	0.092592593
map 05231	Choline metabolism in cancer	0.268227262	3.99483E-05	0.000303137	0.363636364
map 00564	Glycerophospholipid metabolism	1.267983419	5.44277E-05	0.000390065	0.134615385
map 04068	FoxO signaling pathway	0.121921483	6.95244E-05	0.000472034	0.6
map 01100	Metabolic pathways	65.88636918	9.23119E-05	0.000595411	0.025166543
map 00630	Glyoxylate and dicarboxylate metabolism	1.511826384	0.000171042	0.001050688	0.112903226
map 00190	Oxidative phosphorylation	0.390148744	0.000204512	0.001149292	0.25
map 04216	Ferroptosis	0.707144599	0.000204912	0.001149292	0.172413793
map 04924	Renin secretion	0.414533041	0.00026349	0.001359611	0.235294118
map 04964	Proximal tubule bicarbonate reclamation	0.414533041	0.00026349	0.001359611	0.235294118
map 04931	Insulin resistance	0.463301634	0.000416546	0.00206671	0.210526316
map 05012	Parkinson disease	0.48768593	0.000513007	0.002451034	0.2
map 04727	GABAergic synapse	0.219458669	0.000551859	0.002542494	0.333333333
map 04152	AMPK signaling pathway	0.536454523	0.000751888	0.00334244	0.181818182
map 04022	cGMP-PKG signaling pathway	0.243842965	0.000777312	0.00334244	0.3
map 04714	Thermogenesis	0.56083882	0.000896782	0.003731772	0.173913043
map 00052	Galactose metabolism	1.12167764	0.001825222	0.007357924	0.108695652
map 04150	mTOR signaling pathway	0.097537186	0.002198433	0.008593874	0.5
map 00620	Pyruvate metabolism	0.755913192	0.002830833	0.010740513	0.129032258
map 05131	Shigellosis	0.390148744	0.003333451	0.012286147	0.1875
map 01523	Antifolate resistance	0.414533041	0.003991299	0.014302156	0.176470588
map 01210	2-Oxocarboxylic acid metabolism	3.267495733	0.004212296	0.014686113	0.059701493
map 00061	Fatty acid biosynthesis	1.414289198	0.00508542	0.017263662	0.086206897
map 04918	Thyroid hormone synthesis	0.512070227	0.007380826	0.024413503	0.142857143
map 04925	Aldosterone synthesis and secretion	0.536454523	0.008427501	0.02717869	0.136363636
map 00290	Valine, leucine and isoleucine biosynthesis	0.56083882	0.009557109	0.027345439	0.130434783
map 00220	Arginine biosynthesis	0.56083882	0.009557109	0.027345439	0.130434783
map 04724	Glutamatergic synapse	0.195074372	0.009751087	0.027345439	0.25
map 04211	Longevity regulating pathway	0.195074372	0.009751087	0.027345439	0.25
map 04740	Olfactory transduction	0.195074372	0.009751087	0.027345439	0.25
map 05032	Morphine addiction	0.195074372	0.009751087	0.027345439	0.25
map 04024	cAMP signaling pathway	0.609607413	0.012070106	0.033128589	0.12
map 05034	Alcoholism	0.243842965	0.015279969	0.041064916	0.2
map 00310	Lysine degradation	1.316752012	0.020299998	0.053442852	0.074074074
map 00760	Nicotinate and nicotinamide metabolism	1.341136308	0.021575257	0.054211054	0.072727273
map 04742	Taste transduction	0.755913192	0.021700254	0.054211054	0.096774194
map 04721	Synaptic vesicle cycle	0.292611558	0.021852518	0.054211054	0.166666667
map 01200	Carbon metabolism	2.779809802	0.022892225	0.055718812	0.052631579
map 00410	Beta-alanine metabolism	0.780297488	0.023615333	0.056414406	0.09375
map 00471	D-Glutamine and D-glutamate metabolism	0.316995855	0.025502823	0.059815712	0.153846154
map 00400	Phenylalanine, tyrosine and tryptophan biosynthesis	0.829066081	0.027712438	0.06383758	0.088235294
map 04611	Platelet activation	0.341380151	0.029381868	0.065349326	0.142857143
map 04923	Regulation of lipolysis in adipocytes	0.341380151	0.029381868	0.065349326	0.142857143
map 04066	HIF-1 signaling pathway	0.365764448	0.033479574	0.073201103	0.133333333
map 00500	Starch and sucrose metabolism	0.902218971	0.034522413	0.074223189	0.081081081
map 00561	Glycerolipid metabolism	0.926603267	0.036968063	0.078178362	0.078947368
map 00280	Valine, leucine and isoleucine degradation	1.024140454	0.047613224	0.097493744	0.071428571
map 00650	Butanoate metabolism	1.024140454	0.047613224	0.097493744	0.071428571

Legend: KEGG pathways of different metabolites that were altered significantly after GOLPH3L silencing, *p*-values< 0.05

**Fig. 5:**
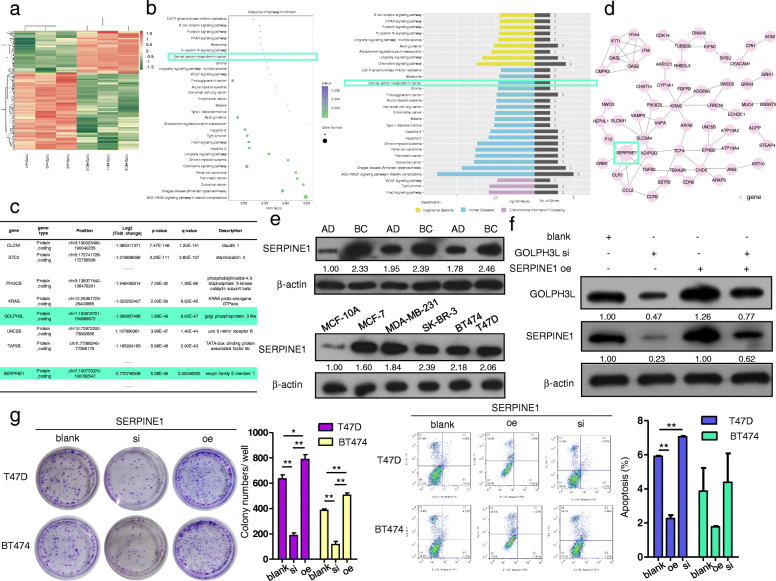
GOLPH3L promotes the expression of SERPINE1, which regulates tumorigenic activities in breast cancer cells. **a** Heat map of transcriptome alterations after silencing GOLPH3L expression in T47D cells; *p*-values are indicated. **b** Bubble diagram of KEGG pathways (left panel) and GO analysis of pathways (right panel) related to transcriptome alteration after the inhibition of GOLPH3L; *p*-values< 0.05 are indicated. **c** The mRNA expression of SERPINE1 was decreased by GOLPH3L silencing, and *p*-values< 0.01 are indicated. d Protein-protein interactions of differentially expressed mRNA of proteins. **e** The protein levels of SERPINE1 were higher in breast cancer tissue samples than in adjacent normal tissues (top panel), and the protein levels of GOLPH3L were higher in breast cancer cell lines than in normal mammary epithelial cells (bottom panel). **f** The expression of GOLPH3L was correlated with SERPINE1 expression, and SERPINE1 overexpression reversed the suppression of SERPINE1 caused by GOLPH3L knockdown. **g** SERPINE1 promoted the proliferation and survival of breast cancer cell lines; *n* = 3, * *p* < 0.05, ** *p* < 0.01

Correspondingly, high-throughput transcriptome sequencing revealed that GOLPH3L expression could markedly alter the expression of 205 genes in breast cancer cells (Fig. 5a and Table 3), and the central carbon metabolism pathway was one of the downregulated pathways in GOLPH3L-knockdown cancer cells (Fig. 5b and Table 4). Because GOLPH3L is located in the Golgi apparatus and may participate in mediating recruitment to Golgi membranes, we predicted that GOLPH3L likely functions by regulating the stability of other proteins involved in tumorigenesis via certain types of glycosylation. Therefore, we chose SERPINE1 as a candidate target through comprehensively analyzing the upregulated tumor suppressor genes and downregulated oncogenes that were related to the central carbon metabolism pathway and had glycosylation as a posttranslational modification (Fig. 5c and d).

**Table 3:** A total of 205 differentially expressed genes were found in T47D cells after GOLPH3L knockdown

Compared group	Upregulated genes	Downregulated genes
NC--si	133	72
	CP, KRT10, PAPSS2, CASP7, UNC5B, AKR1C2, SPRY4, CHST14, FGFR3, SLC6A14, LRRC55, ILF3-AS1, EXOC3-AS1, APLNR, CDK14, AKR1C1, IFI6, OAS2, AXIN2, ZNF702P, MAN1B1-AS1, IFIT1, C2CD4A, AKR1C3, MPV17 L, HABP4, ZCCHC24, PCP4 L1, RASD2, TUB, CRH, STMN3, PALMD, GRIK4, CHD5, FLJ37453, HOXB7, SLC16A10, STEAP4, ARAP3, LOC100132249, SSTR2, SLC5A1, EGR3, ENSG00000261040.2, OASL, ADAM19, TDO2, GAREM2, ENSG00000261071.1, CEACAM1, THEM5, WFDC21P, ADGRA2, ZNF98, PPP1R1B, CDSN, IFI44, SMCO4, SLC22A31, PCDH20, GDF1, COLCA1, C1orf220, B3GNT3, ORM1, ENSG00000254024.1, ADORA1, IFI27, CMPK2, GLTPD2, CCDC177, MYEOV, ENSG00000269962.1, PTCHD1, ENSG00000266795.2, STRA6, RHBDL3, FLJ36000, ANG, RAMP2-AS1, PYDC1, TBXA2R, NELL2, LOC100996419, DLG5-AS1, SLC2A4, GAPDHP43, MYLK-AS1, VGF, EDN2, AMPD3, CNTFR, LOXL4, INSM1, ENSG00000251417.1, KCNB1, ST7-AS1, HMGB1P1, EIF4HP1, GPRC5B, ETV1, ENSG00000233029.3, SNX25P1, ENSG00000273284.1, LINC01023,4-Sep, OGFRP1, TUBB2B, C1S, ENSG00000270964.1, F11-AS1, ENSG00000256982.1, C11orf45, ENSG00000261693.1, F10, ENSG00000236514.1, ZNF663P, TOLLIP-AS1, ALOX12B, TNFAIP8 L3, UNC5CL, VANGL2, ENSG00000269289.1, FRMPD3, PPP1R14BP3, LINC00865, USH1G, ACOT11, HEPHL1, ATP13A4, MUC4, NKX1–2	CLDN1, STC2, FAM65C, CRIM1, PIK3CB, CNOT6, VAPA, REEP3, PON2, AVL9, TMEM45B, VAMP3, KRAS, GOLPH3L, TAF9B, ATP13A2, OLR1, EFNB2, LMBRD1, MAP 6D1, TGFB2, ANXA3, PHACTR1, SHISA2, SH3RF1, USP2, POLR3G, ANKFN1, FSIP2, SAP30 L-AS1, TGFB2-OT1, GRIK1, ACPP, SYNPO, BCHE, ABCC13, PDZK1, DNAH5, SCART1, CCL2, SYBU, ECHDC1, ENSG00000260037.1, LOC100288911, ENSG00000265242.1, SERPINE1, ADIPOQ, ENSG00000254343.2, NWD2, CCR2, KIF5C, OTOR, TCF4, GPR183, SNORD12B, LINC00312, MUM1 L1, TAB3-AS1, CYP1A1, ENSG00000267896.1, PSG9, SPOCK2, ENSG00000269680.1, SLCO2A1, ADM, ENSG00000255142.1, PLAC4, ENSG00000257193.1, ENSG00000231868.1, CRYGS, IL16, ENSG00000236933.1

Legends: KEGG pathways of different metabolites that were altered significantly after GOLPH3L silencing, *p*-values< 0.05

**Table 4:** KEGG pathways involved in GOLPH3L-silenced breast cancer cells

Database	ID	***P***-Value	Corrected ***P***-Value	Genes
KEGG PATHWAY	hsa04933	5.42713E-07	7.32662E-05	CCL2;PIK3CB;SERPINE1;KRAS;TGFB2
KEGG PATHWAY	hsa05142	2.07617E-05	0.001401414	CCL2;PIK3CB;TGFB2;SERPINE1
KEGG PATHWAY	hsa05210	0.000126423	0.003878901	PIK3CB;TGFB2;KRAS
KEGG PATHWAY	hsa05212	0.000151007	0.003878901	PIK3CB;TGFB2;KRAS
KEGG PATHWAY	hsa05211	0.000157602	0.003878901	PIK3CB;TGFB2;KRAS
KEGG PATHWAY	hsa04062	0.00018996	0.003878901	CCL2;PIK3CB;KRAS;CCR2
KEGG PATHWAY	hsa05220	0.000201128	0.003878901	PIK3CB;TGFB2;KRAS
KEGG PATHWAY	hsa04211	0.000412618	0.006962933	PIK3CB;KRAS;ADIPOQ
KEGG PATHWAY	hsa05160	0.001101508	0.01518735	PIK3CB;KRAS;CLDN1
KEGG PATHWAY	hsa04068	0.001124989	0.01518735	PIK3CB;TGFB2;KRAS
KEGG PATHWAY	hsa04530	0.001247139	0.015305797	VAPA;KRAS;CLDN1
KEGG PATHWAY	hsa05161	0.001431726	0.016106915	PIK3CB;TGFB2;KRAS
KEGG PATHWAY	hsa04960	0.001705229	0.017708152	PIK3CB;KRAS
KEGG PATHWAY	hsa04360	0.002413822	0.022159731	EFNB2;PIK3CB;KRAS
KEGG PATHWAY	hsa04930	0.002525744	0.022159731	PIK3CB;ADIPOQ
KEGG PATHWAY	hsa05144	0.002626339	0.022159731	CCL2;TGFB2
KEGG PATHWAY	hsa05213	0.00293929	0.023341421	PIK3CB;KRAS
KEGG PATHWAY	hsa05223	0.003382408	0.024852551	PIK3CB;KRAS
KEGG PATHWAY	hsa05221	0.003497766	0.024852551	PIK3CB;KRAS
KEGG PATHWAY	hsa05205	0.003682986	0.024860155	PIK3CB;TGFB2;KRAS
KEGG PATHWAY	hsa04370	0.003977354	0.025568702	PIK3CB;KRAS
KEGG PATHWAY	hsa04213	0.004355952	0.026010475	PIK3CB;KRAS
KEGG PATHWAY	hsa05214	0.004485724	0.026010475	PIK3CB;KRAS
KEGG PATHWAY	hsa05230	0.004750595	0.026010475	PIK3CB;SERPINE1
KEGG PATHWAY	hsa04664	0.004885683	0.026010475	PIK3CB;KRAS
KEGG PATHWAY	hsa05218	0.005301493	0.026010475	PIK3CB;KRAS
KEGG PATHWAY	hsa04917	0.005443594	0.026010475	PIK3CB;KRAS
KEGG PATHWAY	hsa03320	0.005443594	0.026010475	OLR1;ADIPOQ
KEGG PATHWAY	hsa04662	0.005587435	0.026010475	PIK3CB;KRAS
KEGG PATHWAY	hsa01521	0.006800173	0.030390563	PIK3CB;KRAS
KEGG PATHWAY	hsa05166	0.006978574	0.030390563	PIK3CB;TGFB2;KRAS
KEGG PATHWAY	hsa04060	0.007424504	0.031322125	CCL2;TGFB2;CCR2
KEGG PATHWAY	hsa04012	0.007950284	0.032246156	PIK3CB;KRAS
KEGG PATHWAY	hsa05215	0.008121254	0.032246156	PIK3CB;KRAS
KEGG PATHWAY	hsa05323	0.008468143	0.032662838	CCL2;TGFB2
KEGG PATHWAY	hsa01522	0.009548044	0.034763406	PIK3CB;KRAS
KEGG PATHWAY	hsa04914	0.009733696	0.034763406	PIK3CB;KRAS
KEGG PATHWAY	hsa04915	0.009920954	0.034763406	PIK3CB;KRAS
KEGG PATHWAY	hsa05146	0.010109813	0.034763406	PIK3CB;TGFB2
KEGG PATHWAY	hsa05231	0.010300268	0.034763406	PIK3CB;KRAS
KEGG PATHWAY	hsa04066	0.010685948	0.035185438	PIK3CB;SERPINE1
KEGG PATHWAY	hsa04660	0.011077952	0.035607703	PIK3CB;KRAS
KEGG PATHWAY	hsa04668	0.012085376	0.037712623	CCL2;PIK3CB
KEGG PATHWAY	hsa04725	0.012291521	0.037712623	PIK3CB;KRAS
KEGG PATHWAY	hsa04919	0.013777442	0.039775364	PIK3CB;KRAS
KEGG PATHWAY	hsa04670	0.013777442	0.039775364	PIK3CB;CLDN1
KEGG PATHWAY	hsa05145	0.013995787	0.039775364	PIK3CB;TGFB2
KEGG PATHWAY	hsa04722	0.014215636	0.039775364	PIK3CB;KRAS
KEGG PATHWAY	hsa04071	0.014436984	0.039775364	PIK3CB;KRAS
KEGG PATHWAY	hsa04152	0.015337266	0.041410618	PIK3CB;ADIPOQ
KEGG PATHWAY	hsa04380	0.016969364	0.044918905	PIK3CB;TGFB2
KEGG PATHWAY	hsa04650	0.01769056	0.045927416	PIK3CB;KRAS
KEGG PATHWAY	hsa04910	0.018672129	0.047302645	PIK3CB;KRAS
KEGG PATHWAY	hsa04210	0.018921058	0.047302645	PIK3CB;KRAS
KEGG PATHWAY	hsa04550	0.019423126	0.047674946	PIK3CB;KRAS
KEGG PATHWAY	hsa04072	0.019930779	0.048047414	PIK3CB;KRAS
KEGG PATHWAY	hsa05200	0.021609077	0.05050568	PIK3CB;TGFB2;KRAS
KEGG PATHWAY	hsa04932	0.021751022	0.05050568	PIK3CB;ADIPOQ
KEGG PATHWAY	hsa04390	0.022551551	0.05050568	TGFB2;SERPINE1
KEGG PATHWAY	hsa04150	0.022551551	0.05050568	PIK3CB;KRAS
KEGG PATHWAY	hsa04145	0.022821085	0.05050568	VAMP3;OLR1
KEGG PATHWAY	hsa04921	0.023637696	0.051469176	PIK3CB;KRAS
KEGG PATHWAY	hsa05164	0.028783631	0.06167921	CCL2;PIK3CB
KEGG PATHWAY	hsa05168	0.031818708	0.067117587	CCL2;TAF9B
KEGG PATHWAY	hsa04977	0.036402182	0.075604532	LMBRD1
KEGG PATHWAY	hsa05169	0.037582935	0.076394178	POLR3G;PIK3CB
KEGG PATHWAY	hsa05203	0.037914147	0.076394178	PIK3CB;KRAS
KEGG PATHWAY	hsa04015	0.039924992	0.079262851	PIK3CB;KRAS
KEGG PATHWAY	hsa04810	0.041287714	0.08078031	PIK3CB;KRAS
KEGG PATHWAY	hsa04320	0.042104357	0.081201261	KRAS
KEGG PATHWAY	hsa05216	0.04352471	0.082758252	KRAS
KEGG PATHWAY	hsa04014	0.045835719	0.085941973	PIK3CB;KRAS
KEGG PATHWAY	hsa00640	0.047773358	0.0871541	ECHDC1
KEGG PATHWAY	hsa03020	0.047773358	0.0871541	POLR3G

Legends: KEGG pathways of different metabolites that were altered significantly after GOLPH3L silencing, *p*-values< 0.05

### GOLPH3L stabilizes p53-induced SERPINE1 expression in breast Cancer cells is positively correlated with increased glycolysis

**Fig. 6:**
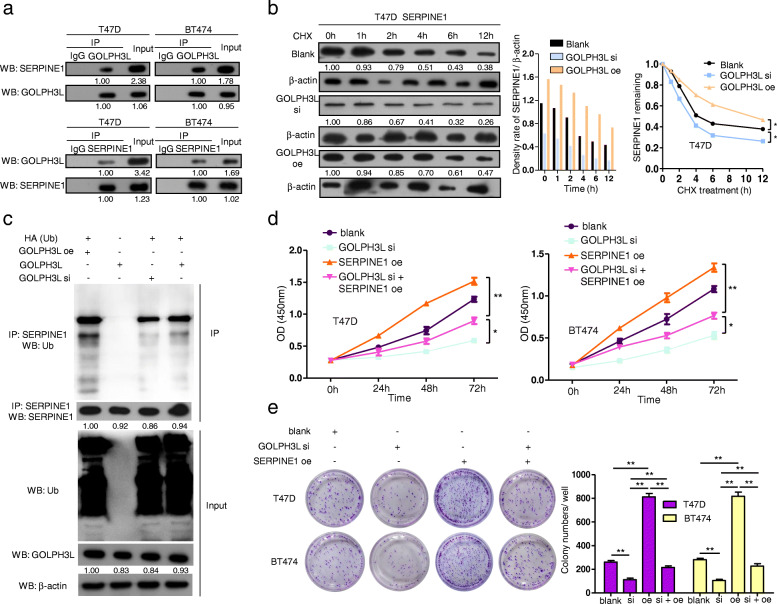
GOLPH3L interacts with SERPINE1 by stabilizing its expression and promotes its tumorigenic activity. **a** GOLPH3L was confirmed to interact with SERPINE1 in T47D and BT474 cells with a Co-IP assay. **b** The knockdown of GOLPH3L in T47D cells reduced the stability of SERPINE1, while overexpressing GOLPH3L stabilized SERPINE1; *p*-values< 0.05 are indicated. **c** Expression of GOLPH3L was correlated with the ubiquitination levels of SERPINE1. T47D cells with GOLPH3L siRNA and/or overexpression were subjected to immunoprecipitation and Western blot analyses. **d** The overexpression of SERPINE1 partially reversed the proliferative defects induced by GOLPH3L suppression. The cell number was determined with CCK-8 assay, *n* = 3. * *p* < 0.05, ** *p* < 0.01. **e** SERPINE1 expression rescued the inhibition of tumorigenesis caused by GOLPH3L suppression. Representative images of colonies revealed by crystal violet staining and quantification of colony numbers of each group, *n* = 3. * *p* < 0.05, ** *p* < 0.01

Published data [28] and our KEGG analysis results (Figure S4c) indicated that the p53 signaling pathway regulates SERPINE1 and therefore suppresses tumor cell proliferation, invasion and migration, whereas it promotes cell apoptosis. To assess the role of SERPINE1 in breast cancer, we examined the expression level of SERPINE1. When compared to those in the tumor-adjacent normal tissues and normal breast epithelial cell line MCF-10A, the expression levels of SERPINE1 were increased in breast cancer tissues and breast cancer cell lines (Fig. 5e). In addition, the protein levels of SERPINE1 were correlated with the protein levels of GOLPH3L in breast cancer cells, supporting the notion that GOLPH3L may regulate the expression of SERPINE1 (Fig. 5f). Moreover, the expression of SERPINE1 promoted breast tumorigenesis (Fig. 5g). Using a coimmunoprecipitation (Co-IP) assay, we confirmed the interaction between GOLPH3L and SERPINE1 in breast cancer cells (Fig. 6a). To test whether GOLPH3L can stabilize SERPINE1, the alteration of the expression of GOLPH3L significantly affected the half-life of SERPINE1 in a breast cancer cell line, indicating that GOLPH3L contributes to the stabilization of SERPINE1 (Fig. 6b). Subsequently, we altered the expression of GOLPH3L in T47D cells, and the IP results showed increased ubiquitination of SERPINE1 in the presence of GOLPH3L (Fig. 6c). Furthermore, the overexpression of SERPINE1 reversed the antitumor activities induced by the suppression of GOLPH3L in breast cancer cell lines (Fig. 6d and e). Therefore, GOLPH3L promotes glucose metabolism in breast cancer and is conducive to SERPINE1 stabilization.

## Discussion

Breast cancer cells are produced by mutations in the DNA of normal breast cells, genetics and other factors, such as diet and exercise. Lifestyle habits can also be the cause of DNA mutations. Some DNA mutations are heritable, but the vast majority of DNA changes are acquired, and such acquired mutations occur only in breast cancer cells [29]. Mutated DNA causes corresponding changes in genes that regulate cell proliferation and apoptosis, which can lead to uncontrolled proliferation and apoptosis, thus promoting the development of tumors. According to the New York Cancer Institute [30], targeted therapeutics can be categorized according to their targets: those that target the regulatory mechanism of tumor formation, those that target the tumor microenvironment, those that target the tumor immune system, those that target tumor markers and those that target tumor stem cells. Dysregulation of targets that regulate tumor formation can stimulate a series of downstream signaling pathways, resulting in abnormal division, growth, metabolism, cell environment and angiogenesis of tumor cells compared with normal cells.

Golgi phosphoprotein 3-like (GOLPH3L) is a prognostic biomarker of cervical cancer [24] and ovarian cancer [25] and may be a mitochondrial biogenesis marker in breast cancer metabolism [31]. However, the roles of GOLPH3L in breast tumorigenesis remain unclear. SERPINE1 promotes breast cancer cell metastasis [32] and glycolytic metabolism in triple-negative breast cancer (TNBC) [33] and participates in EGFR signaling [34]. We discovered that GOLPH3L promotes glucose metabolism in breast cancer cells by stabilizing SERPINE1, which is regulated by p53 transcription [35]. In this context, we demonstrate that silencing the GOLPH3L gene suppresses breast tumorigenesis and glucose metabolism, while overexpression of GOLPH3L promotes breast tumorigenesis. Therefore, GOLPH3L-mediated stabilization of SERPINE1 could represent an important oncogenic pathway in the glucose metabolism of breast cancer. Our findings suggest that GOLPH3L functions as an oncogene in breast cancer by promoting cellular proliferation and migration and suppressing apoptosis. Considering the tumorigenic role of GOLPH3L, we also investigated the upstream regulators of GOLPH3L. Fortunately, we identified that miR-1185-2-3p directly targets the sites within the 3′-UTR of GOLPH3L and functionally suppresses the expression of GOLPH3L. In support of this notion, we demonstrated that miR-1185-2-3p inhibits breast tumorigenesis by suppressing GOLPH3L expression. This is the first time that miR-1185-2-3p has been identified as a regulator in cancer development. Therefore, it will be important to identify the pathway related to miR-1185-2-3p.

The treatment and prognosis of breast cancer differs according to the expression of different molecular makers such as estrogen receptor (ER), progesterone receptor (PR) and human epidermal growth factor 2 (HER2). HER2+ breast cancers tend to grow and spread more aggressively. Different types of drugs such as monoclonal antibodies and kinase inhibitors have been developed to target the HER2 protein [36, 37]. Approximately 2 of 3 breast cancers are hormone receptor-positive (ER+ or PR+). Treatment with hormone therapy is often helpful in these cases, and certain targeted therapy drugs, such as CDK4/6 inhibitors [13], mTOR inhibitor (Everolimus) and PI3K inhibitor (Alpelisib) [38], can increase the efficacy of hormone therapy. Among all types of breast cancer, triple-negative breast cancer (TNBC) has the worst prognosis due to the lack of an effective target. Moreover, over 30% of breast cancer patients may suffer recurrence. In terms of metastatic breast cancer, systemic therapy usually shows unsatisfactory treatment effects. Therefore, it is of great clinical significance to discover new therapeutic targets for breast cancer. Oncogenic proteins such as hypoxia-inducible factor, Myc, p53 and PI3K/Akt/mTOR pathway proteins, which are involved in metabolic reprogramming [39], may serve as candidate anticancer agents. Several preclinical trials have shown effectiveness of an inhibitor targeting the glycolytic pathway [40]. Our discovery provides a mechanistic link between miR-1185-2-3p, GOLPH3L and SERPINE1; this pathway plays a significant role in glucose metabolism in breast cancer and may serve as a novel therapeutic target for breast cancer.

## Conclusions

We discovered a functional pathway linking miR-1185-2-3p, GOLPH3L and SERPINE1 that plays a significant role in glucose metabolism in breast cancer and provides new therapeutic targets for breast cancer treatment.

## Supplementary Information

**Additional file 1:Supplementary Figure 1.** GOLPH3L regulated the tumorigenic activities of BT474 cells. (A) Knockdown of GOLPH3L in MCF-10A, BT474 and T47D cells with five distinct siRNAs. (B) The overexpression (oe) of GOLPH3L in MCF-10A, BT474 and T47D cells. (C) The expression of GOLPH3L in BT474 cells promotes their proliferation, *n* = 3, * *p* < 0.05, ** *p* < 0.01. (D) GOLPH3L expression suppresses the apoptosis of BT474 cells, *n* = 3, ** *p* < 0.01. (E) Knockdown of GOLPH3L inhibits the migration of BT474 cells using a transwell assay, *n* = 3, * *p* < 0.05, ** *p* < 0.01. (F) The suppression of GOLPH3L significantly blocks the BT474 cell cycle at G0/ G1 phase, *n* = 3, * *p* < 0.05, ** *p* < 0.01. **Supplementary Figure 2**. miR-1185-2-3p negatively regulates the tumorigenesis of BT474 cells. (A) The predicted miRNAs which were most likely to regulate with GOLPH3L. (B) The overexpression of miR-1185-2-3p was achieved with miRNA mimics and inhibition miR-1185-2-3p was achieved with miRNA inhibitor in T47D and BT474, *n* = 3, ** *p* < 0.01. (C) The overexpression of miR-1185-2-3p inversely correlated with the proliferation of BT474 cells, *n* = 3, ** *p* < 0.01. (D) The overexpression of miR-1185-2-3p promoted the apoptosis of BT474 cells, *n* = 3, * *p* < 0.05. (E) miR-1185-2-3p overexpression could significantly inhibited the migration of BT474 cells, *n* = 3, * *p* < 0.05. (F) The overexpression of miR-1185-2-3p could block the cell cycle at G0/ G1 phase, *n* = 3, * *p* < 0.05. **Supplementary Figure 3**. miR-1185-2-3p overexpression partially reversed the tumorigenesis induced by GOLPH3L overexpression. (A) The overexpression of miR-1185-2-3p inversely correlated with the up-regulation of proliferation induced by GOLPH3L overexpression in T47D cells, *n* = 3, * *p* < 0.05, ** *p* < 0.01. (B) miR-1185-2-3p partially reversed the apoptosis induced by GOLPH3L overexpression in T47D cells, *n* = 3. * *p* < 0.05. (C) Overexpressed miR-1185-2-3p inhibited the up-regulation of migration caused by GOLPH3L up-regulation using transwell assay, *n* = 3. * *p* < 0.05. (D) The overexpression of miR-1185-2-3p affected the cell cycle regulation by GOLPH3L, *n* = 3, * *p* < 0.05, ** *p* < 0.01.**Supplementary Figure 4.** p53 pathway promotes the transcription of SERPINE1. (A) Volcano plots of fold changes and *p*-values of altered metabolites after GOLPH3L suppression in the Positive Ion Mode (top panel) and Negative Ion Mode (bottom panel). (B) Volcano plots of fold changes and *p*-values of RNA-seq data after silencing GOLPH3L expression in T47D cells. (C) STRING analysis predicted protein-protein network indicating that SERPINE1 interacted with p53 (top panel) and the role of SERPINE1 in p53 signaling pathway after GOLPH3L knockdown in T47D cells (bottom panel).

## Data Availability

The datasets used and/or analyzed during the current study are available from the corresponding author on reasonable request.

## Abbreviations

GOLPH3L: Golgi phosphoprotein 3-like
SERPINE1: Serpin family E member 1
EGFR: Epidermal growth factor receptor
EdU: 5-ethynyl-2′- deoxyuridine
