# Supplementary material for: The miR-1185-2-3p—GOLPH3L pathway promotes glucose metabolism in breast cancer by stabilizing p53-induced SERPINE1
Source: J Exp Clin Cancer Res. 2021 Jan 28;40:47. doi: 10.1186/s13046-020-01767-9 (PMC7842049; doi:10.1186/s13046-020-01767-9)
Supplement: Supplementary file 1 — Additional file 1:Supplementary Figure 1. GOLPH3L regulated the tumorigenic activities of BT474 cells. (A) Knockdown of GOLPH3L in MCF-10A, BT474 and T47D cells with five distinct siRNAs. (B) The overexpression (oe) of GOLPH3L in MCF-10A, BT474 and T47D cells. (C) The expression of GOLPH3L in BT474 cells promotes their proliferation, n = 3, * p < 0.05, ** p < 0.01. (D) GOLPH3L expression suppresses the apoptosis of BT474 cells, n = 3, ** p < 0.01. (E) Knockdown of GOLPH3L inhibits the migration of BT474 cells using a transwell assay, n = 3, * p < 0.05, ** p < 0.01. (F) The suppression of GOLPH3L significantly blocks the BT474 cell cycle at G0/ G1 phase, n = 3, * p < 0.05, ** p < 0.01. Supplementary Figure 2. miR-1185-2-3p negatively regulates the tumorigenesis of BT474 cells. (A) The predicted miRNAs which were most likely to regulate with GOLPH3L. (B) The overexpression of miR-1185-2-3p was achieved with miRNA mimics and inhibition miR-1185-2-3p was achieved with miRNA inhibitor in T47D and BT474, n = 3, ** p < 0.01. (C) The overexpression of miR-1185-2-3p inversely correlated with the proliferation of BT474 cells, n = 3, ** p < 0.01. (D) The overexpression of miR-1185-2-3p promoted the apoptosis of BT474 cells, n = 3, * p < 0.05. (E) miR-1185-2-3p overexpression could significantly inhibited the migration of BT474 cells, n = 3, * p < 0.05. (F) The overexpression of miR-1185-2-3p could block the cell cycle at G0/ G1 phase, n = 3, * p < 0.05. Supplementary Figure 3. miR-1185-2-3p overexpression partially reversed the tumorigenesis induced by GOLPH3L overexpression. (A) The overexpression of miR-1185-2-3p inversely correlated with the up-regulation of proliferation induced by GOLPH3L overexpression in T47D cells, n = 3, * p < 0.05, ** p < 0.01. (B) miR-1185-2-3p partially reversed the apoptosis induced by GOLPH3L overexpression in T47D cells, n = 3. * p < 0.05. (C) Overexpressed miR-1185-2-3p inhibited the up-regulation of migration caused by GOLPH3L up-regulation us [file 13046_2020_1767_MOESM1_ESM.pdf]

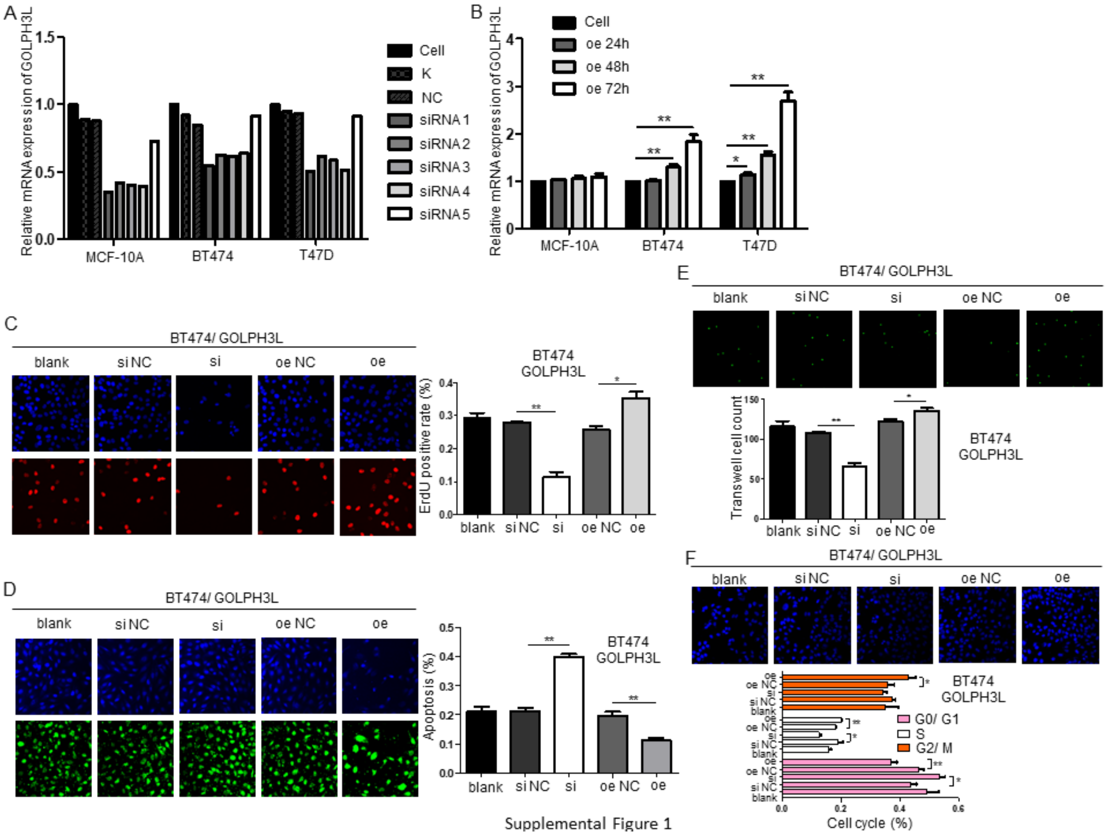

Supplementary Figure 1. GOLPH3L regulated the tumorigenic activities of BT474 cells. (A) Knockdown of GOLPH3L in MCF-10A, BT474 and T47D cells with five distinct siRNAs. (B) The overexpression (oe) of GOLPH3L in MCF-10A, BT474 and T47D cells. (C) The expression of GOLPH3L in BT474 cells promotes their proliferation, n=3, * p <0.05, ** p <0.01. (D) GOLPH3L expression suppresses the apoptosis of BT474 cells, n=3, ** p <0.01. (E) Knockdown of GOLPH3L inhibits the migration of BT474 cells using a transwell assay, n=3, * p <0.05, ** p <0.01. (F) The suppression of GOLPH3L significantly blocks the BT474 cell cycle at G0/ G1 phase, n=3, * p <0.05, ** p <0.01.

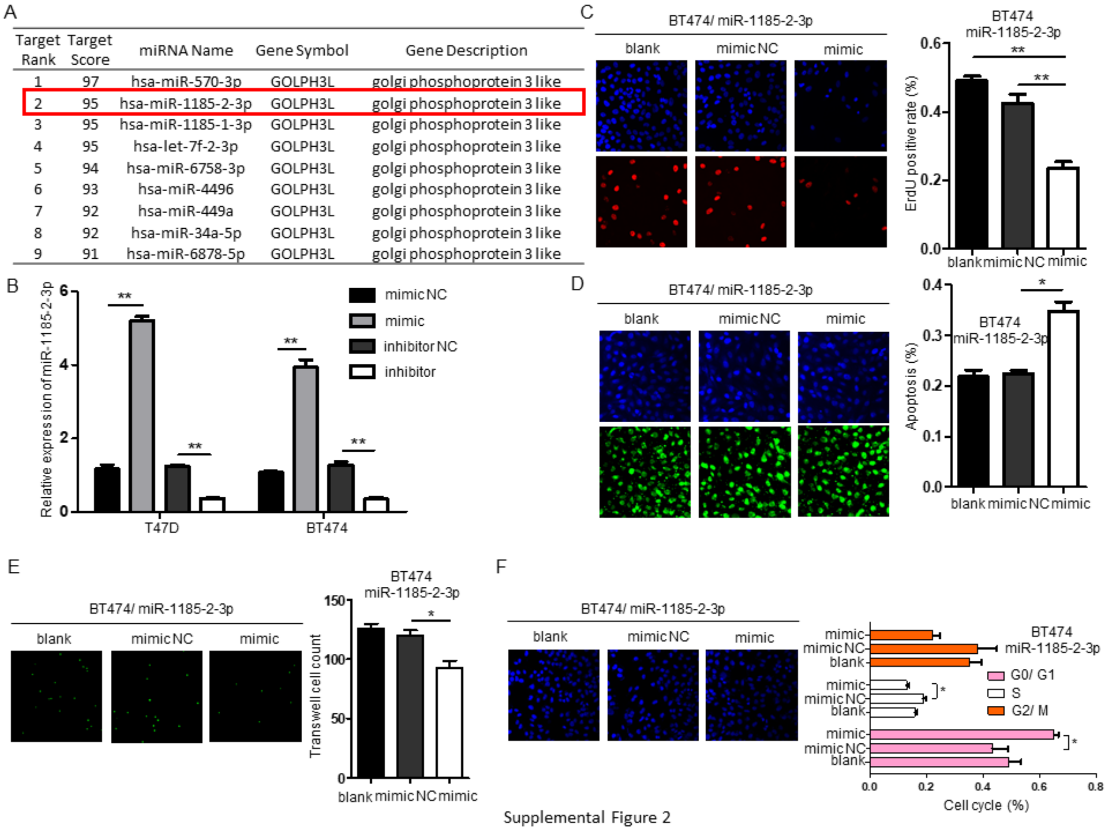

Supplemental Figure 2

Supplementary Figure 2. miR-1185-2-3p negatively regulates the tumorigenesis of BT474 cells. (A) The predicted miRNAs which were most likely to regulate with GOLPH3L. (B) The overexpression of miR-1185-2-3p was achieved with miRNA mimics and inhibition miR-1185-2-3p was achieved with miRNA inhibitor in T47D and BT474, n=3, ** p <0.01. (C) The overexpression of miR-1185-2-3p inversely correlated with the proliferation of BT474 cells, n=3, ** p <0.01. (D) The overexpression of miR-1185-2-3p promoted the apoptosis of BT474 cells, n=3, * p <0.05. (E) miR-1185-2-3p overexpression could significantly inhibited the migration of BT474 cells, n=3, * p <0.05. (F) The overexpression of miR-1185-2-3p could block the cell cycle at G0/ G1 phase, n=3, * p <0.05.

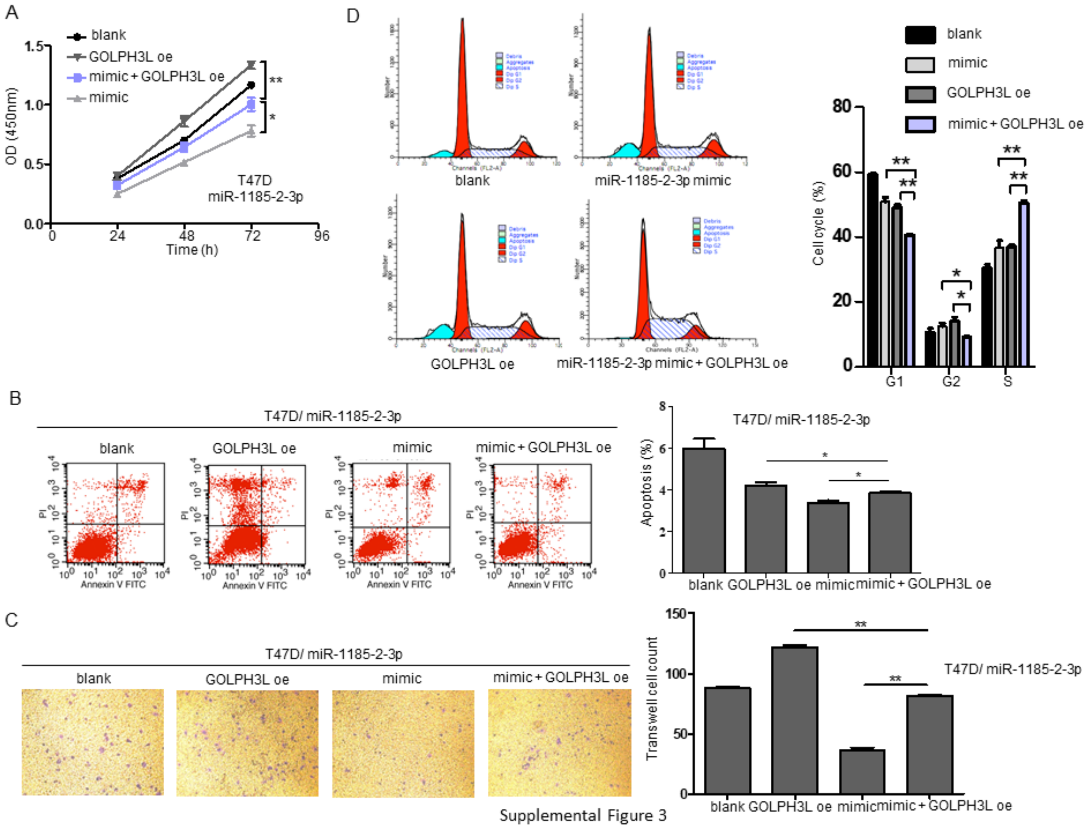

Supplementary Figure 3. miR-1185-2-3p overexpression partially reversed the tumorigenesis induced by GOLPH3L overexpression. (A) The overexpression of miR-1185-2-3p inversely correlated with the up-regulation of proliferation induced by GOLPH3L overexpression in T47D cells, n=3, * p <0.05, ** p <0.01. (B) miR-1185-2-3p partially reversed the apoptosis induced by GOLPH3L overexpression in T47D cells, n=3. * p <0.05. (C) Overexpressed miR-1185-2-3p inhibited the up-regulation of migration caused by GOLPH3L up-regulation using transwell assay, n=3. * p <0.05. (D) The overexpression of miR-1185-2-3p affected the cell cycle regulation by GOLPH3L, n=3, * p <0.05, ** p <0.01.

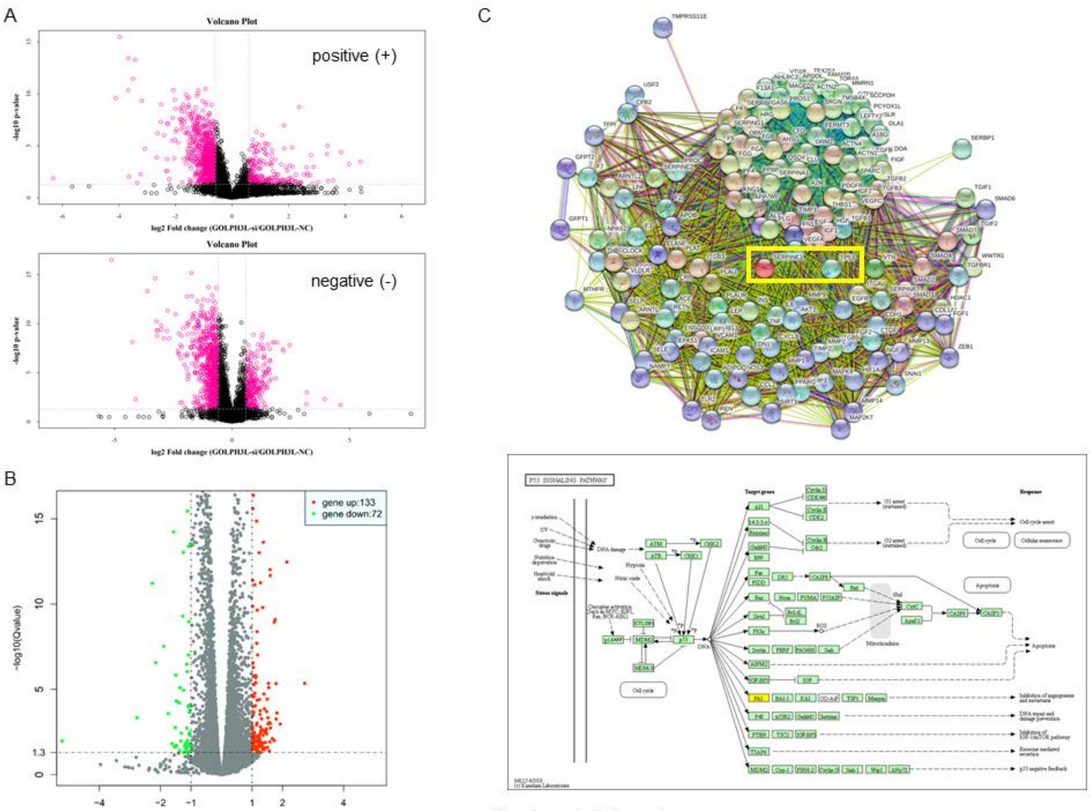

Supplemental Figure 4

Supplementary Figure 4. p53 pathway promotes the transcription of SERPINE1. (A) Volcano plots of fold changes and p-values of altered metabolites after GOLPH3L suppression in the Positive Ion Mode (top panel) and Negative Ion Mode (bottom panel). (B) Volcano plots of fold changes and p-values of RNA-seq data after silencing GOLPH3L expression in T47D cells. (C) STRING analysis predicted protein-protein network indicating that SERPINE1 interacted with p53 (top panel) and the role of SERPINE1 in p53 signaling pathway after GOLPH3L knockdown in T47D cells (bottom panel).
